# Faecal incontinence—a comprehensive review

**DOI:** 10.3389/fsurg.2024.1340720

**Published:** 2024-02-01

**Authors:** Eloise Dexter, Josephine Walshaw, Hannah Wynn, Safaa Dimashki, Alex Leo, Ian Lindsey, Marina Yiasemidou

**Affiliations:** ^1^ Colorectal Surgery, Liverpool University Hospitals NHS Foundation Trust, Liverpool, United Kingdom; ^2^ Leeds Institute of Medical Research, St James’ University Hospital, University of Leeds, Leeds, United Kingdom; ^3^ Department of Health Research, University of York, York, United Kingdom; ^4^ Upper Gastrointestinal Surgery, York and Scarborough Teaching Hospitals NHS Foundation Trust, York, United Kingdom; ^5^ Plastic Surgery, Mid Yorkshire Teaching NHS Trust, Wakefield, United Kingdom; ^6^ Colorectal Surgery, The Royal London Hospital, Barts Health NHS Trust, London, United Kingdom; ^7^ Colorectal Surgery, Oxford University Hospitals, Oxford, United Kingdom

**Keywords:** faecal incontinence, fecal incontinence, conservative management, surgical management, sphincter injury

## Abstract

**Introduction:**

Faecal incontinence (FI) is a distressing and often stigmatizing condition characterised as the recurrent involuntary passage of liquid or solid faeces. The reported prevalence of FI exhibits considerable variation, ranging from 7 to 15% in the general population, with higher rates reported among older adults and women. This review explores the pathophysiology mechanisms, the diagnostic modalities and the efficiency of treatment options up to date.

**Methods:**

A review of the literature was conducted to identify the pathophysiological pathways, investigation and treatment modalities.

**Result and discussion:**

This review provides an in-depth exploration of the intricate physiological processes that maintain continence in humans. It then guides the reader through a detailed examination of diagnostic procedures and a thorough analysis of the available treatment choices, including their associated success rates. This review is an ideal resource for individuals with a general medical background and colorectal surgeons who lack specialized knowledge in pelvic floor disorders, as it offers a comprehensive understanding of the mechanisms, diagnosis, and treatment of faecal incontinence (FI).

## Background

Faecal incontinence (FI) is a distressing and often stigmatising condition characterised as the recurrent involuntary passage of liquid or solid faeces (1). Anal incontinence (AI) is FI encompassing the inclusion of flatus and mucus into the definition (2). The reported prevalence of AI exhibits considerable variation, ranging from 7 to 15% in the general population, with higher rates reported among older adults (1, 3, 4) and women (5). Three main subtypes have been delineated: (1) Passive incontinence, characterised by the involuntary passage of stool or gas without conscious awareness. (2) Urge incontinence, whereby faecal contents are expelled despite efforts to prevent such occurrences and (3) faecal seepage, entailing the leakage of stool following otherwise typical bowel movements (5). The extent of the incontinence spans from occasional episodic faecal leakage to complete loss of bowel control (3). Irrespective of its severity, FI poses a considerable physical, psychological, and social challenge for those affected, often leading to a diminished quality of life (6).

Due to its complex origins, faecal incontinence (FI) requires a thorough grasp of its causes, physical processes, methods for diagnosis, and treatment methods. This all-encompassing review aims to delve into the present level of understanding regarding FI. By attaining a well-rounded comprehension of FI, medical professionals can play a role in enhancing patient results and their overall quality of life.

## Physiology of bowel control

Bowel control is a complex physiological process that involves intricate coordination between the nervous system, the gastrointestinal system and pelvic floor muscles. FI can result from various physiological abnormalities and disruptions in the complex process of bowel control.

### Anal sphincter muscles

The anal sphincter muscles, the involuntary internal anal sphincter (IAS) made of smooth muscle, and the voluntary external anal sphincter (EAS) made of skeletal muscle, are vital for maintaining continence. The IAS remains contracted at rest, preventing stool leakage, while the EAS adds extra control. It can be consciously contracted for increased anal pressure during activities like coughing. Coordination between these muscles and the puborectalis muscle is crucial for continence. Weakness or damage to these muscles, including but not limited to obstetric trauma during vaginal delivery, anorectal surgical procedures including anal dilation, haemorrhoidectomy, fistulotomy and sphincterotomy can result in leakage (7).

### Pelvic floor muscles

The pelvic floor muscles provide support to the pelvic organs and contribute to continence. The puborectalis muscle is a critical component of the pelvic floor, forming a sling around the anorectal junction. This muscle's function is to maintain the angle between the rectum and anal canal, creating a kink that helps prevent involuntary stool leakage (7). During the defecation process, the puborectalis muscle relaxes to straighten the rectal angle, allowing for easier stool passage (7). Impaired coordination between the pelvic floor muscles and the anal sphincter can compromise the ability to maintain continence during activities such as coughing, sneezing, or physical exertion (1).

### Rectal sensation and compliance

The rectum functions as a storage reservoir for faeces. As stool accumulates, it distends the rectal walls, leading to the activation of the rectal mechanoreceptors (1). They detect the stretching and transmit sensory signals to the central nervous system, providing the sensation of rectal fullness and triggering the urge to defecate (1). The rectum also exhibits compliance, allowing it to accommodate faecal material until it is appropriate to initiate defecation. When the rectal volume increases, it triggers the urge to defecate, and coordinated relaxation of the IAS occurs (1).This sensory feedback is essential for recognizing the appropriate time to initiate a bowel movement (1). Alterations in the sensory perception of the rectum, for instance due to neurological disorders affecting the central or peripheral nervous system or the spinal cord, neuropathy secondary to diabetes mellitus (8), can lead to a diminished awareness of rectal filling and urge sensation, resulting in involuntary bowel movements.

### Stool consistency and volume

The texture and quantity of stool are directly linked to gastrointestinal transit time. Typically, loose stools are associated with rapid transit, while constipation is linked to slow gastrointestinal transit and diminished motility (9). Prolonged transit time facilitates increased water absorption from bowel contents. The entry of stool or flatus into the rectum leads to distention and temporary relaxation of the internal sphincter, allowing the highly innervated anal transition zone to sample the contents (10). Subsequent higher centre perception enables additional relaxation of the sphincter complex at an opportune moment for stool passage. Any interference, dysfunction, or overwhelming of this process may result in incontinence (10). Hard stools resulting in a palpable rectal mass have been shown to have a significant correlation with “overflow” faecal soiling (11), whereas the mechanism of loose stools leading to larger quantities of liquid faecal material may overpower the continence mechanism (9).

### Neural control

The coordinated function of the central nervous system (CNS), autonomic nervous system (ANS), and enteric nervous system (ENS) is vital for maintaining continence. The brain processes the sensory information from the rectal mechanoreceptors and makes a conscious decision about when and where to initiate the defecation process. The prefrontal cortex is particularly involved in the voluntary control of defecation. It evaluates the sensory input and decides whether to initiate or suppress the urge to defecate based on various factors, including social norms, personal habits, and environmental cues (1). The CNS also coordinates the relaxation and contraction of the anal sphincters and pelvic floor muscles during the defecation process (1).

The ANS, operating largely involuntarily influences bowel motility and sphincter function. The sympathetic nervous system is responsible for the “fight or flight” response and is generally inhibitory to the digestive process (3). It helps maintain faecal continence by promoting the contraction of the internal anal sphincter and reducing motility in the colon, contributing to the storage of stool (3). Conversely, the parasympathetic nervous system is responsible for the “rest and digest” response, and it plays a vital role in promoting defecation. When the urge to defecate is sensed, the parasympathetic nerves stimulate peristaltic contractions in the colon and rectum, while also relaxing the internal anal sphincter to allow stool to pass (3).

ENS, an intrinsic network of nerves located entirely within the walls of the gastrointestinal tract, regulates bowel movements locally and coordinates defecation. It functions independently but is influenced by both the CNS and ANS (12). The ENS receives input from sensory neurons within the gut walls, detecting factors like stretching of the intestines and the presence of faecal material. It then coordinates local reflexes that control smooth muscle contractions and regulate the opening and closing of the anal sphincters (12). This local control helps ensure that bowel movements occur in a coordinated and timely manner.

The CNS, ANS, and ENS work in tandem to maintain faecal continence and regulate bowel movements. The CNS processes sensory input from the rectum, generating the sensation of the urge to defecate and coordinating voluntary control. The ANS modulates the balance between storage and elimination, while the ENS provides local control within the gut to regulate motility and sphincter function. Nerve damage, often associated with conditions like diabetes or previous pelvic surgeries, can disrupt the communication between the rectum, anal sphincter, and the brain, leading to loss of bowel control (1).

## Causes

Any disruption or dysfunction to the process of bowel control can lead to faecal incontinence. For example, weakness or injury to the anal sphincter muscles can result in the inability to maintain anal closure, while damage to the nerves controlling bowel function can cause impaired rectal sensation or coordination. Structural abnormalities, such as anorectal malformations or rectal prolapse, can also contribute to faecal incontinence. Hence, FI can have various causes, and the underlying factors can differ depending on the age group and individual circumstances (8).

### Acquired structural abnormalities

Acquired structural abnormalities can have significant implications for bowel control and may result in FI. This occurs from various conditions that impact the anatomical integrity of the rectal and anal region. One common cause is obstetric injury, particularly to individuals who have undergone instrument-assisted vaginal delivery or experienced traumatic deliveries involving significant vaginal tears (10, 13). Anorectal surgeries, such as procedures for haemorrhoids, fistulas, or fissures, can also contribute to structural abnormalities that disrupt bowel control (8). Surgical interventions in the anorectal area may result in scarring, nerve damage, or altered sphincter function, affecting the ability to maintain continence (14). Rectal prolapse is another structural abnormality that can lead to faecal incontinence. In this condition, the rectum protrudes through the anus due to impaired rectal closure (14). The eversion of the sensing zone of the anal canal leads to feedback about arriving stool being delayed too late or absent (14). Rectocele, a condition where the rectum bulges into the posterior vaginal wall, can also impact bowel control (8). Over time, the positional instability of pelvic structures and the inadequate start and finish of defecation can lead to a decrease in functional capacity and potentially more frequent and unintended evacuations, in addition to everting the crucial sensing zone of the anal canal, such that feedback about arriving stool comes too later or not all (14). Finally, trauma, such as pelvic fractures resulting from accidents or injuries, can cause damage to the pelvic floor and anal sphincters leading to FI (15).

### Congenital disorders

Congenital disorders can significantly impact bowel control and lead to faecal incontinence from an early age. Anorectal malformations (ARM) encompass a diverse group of congenital defects that affect the development of the anus and rectum (16). Imperforate anus, one of the most common types of ARM, refers to the absence or abnormal location of the anal opening, hindering the normal passage of stool (16). Cloacal defects, another form of ARM, involve the presence of a single common channel for the rectum, vagina, and urinary tract, leading to challenges in bowel and urinary control (16). Another congenital disorder is spina bifida, a neural tube defect, affects the development of the spinal cord and surrounding structures. Severe forms of spina bifida, such as myelomeningocele or meningocele, involve the protrusion of the spinal cord through an opening in the vertebral column (17). Patients commonly display motor and sensory neurological impairments below the affected area. Urinary and faecal incontinence are prevalent issues (17).

### Defecation disorders

Several factors can affect the normal mechanisms of bowel control and cause FI. Chronic or frequent episodes of diarrhoea can have a negative impact on faecal continence. The increased frequency and urgency associated with diarrhoea can overwhelm the anal sphincters' ability to hold stool, resulting in faecal leakage (12). A further cause is faecal impaction causing paradoxical diarrhoea, whereby liquid stool leaks around a large, impacted mass in the rectum (18). This can create an atypical “obstruction” that prevents normal stool passage and results in the involuntary leakage of liquid stool (12). Similarly, prolonged constipation can cause a build-up of hard, impacted stool in the rectum. The stretched rectum can lose its ability to sense fullness, leading to reduced awareness of the need to defecate (19). As a result, the weakened rectal muscles may not be able to generate the force required to expel stool properly, leading to involuntary leakage (19).

Co-existence of constipation and FI is well-known within the elderly and paediatric population, This is known to present as stool withholding behaviour and subsequent overflow in paediatric populations, and faecal impaction with overflow in the elderly population (20). In contrast to this, FI and constipation in adults are often regarded as distinct and separate conditions. Vollebregt et al. studied 4,027 (aged 18–80) patients, referred to a tertiary unit for investigation of refractory faecal incontinence and/or constipation, to assess the frequency in which coexistent diagnosis were made (20). The outcomes were that over 40% of patients who were referred for anorectal physiological investigation had co-existing FI and constipation. Notably, 86.4% of the patients had recognition of only constipation or FI alone, rather than co-existent pathologies when initially referred (20).

Pelvic floor dysfunction is another significant cause of faecal incontinence. Weakened support to the rectum and sphincter complex can lead to inadequate control of motions and FI (21).

Additionally, certain psychological factors, such as severe anxiety, depression, or cognitive impairments, can influence bowel control (8). Emotional and behavioural factors can lead to alterations in gut motility and exacerbate existing bowel problems, contributing to faecal incontinence (19). In some cases, cognitive impairment may lead to difficulties in recognizing the urge to defecate or in communicating the need for assistance, contributing to incontinence (19).

### Neurological disorders

Nerve function plays a vital role in the coordinated control of bowel movements. When nerves supplying the rectum and anus are damaged or dysfunctional, the communication between the rectum and the brain can be disrupted (18). This can lead to a loss of sensation, preventing individuals from detecting rectal fullness or the urge to defecate, and can also impair the signals needed to contract and relax the anal sphincters effectively (1). Neurological disorders and nerve problems can significantly impact bowel control and lead to faecal incontinence.

Pudendal neuropathy, resulting from nerve damage or compression of the pudendal nerve, can arise from various causes, such as radiation therapy, diabetes, or chemotherapy (18). The pudendal nerve plays a critical role in controlling the anal sphincters and pelvic floor muscles, and its dysfunction can lead to impaired coordination and weakness, contributing to faecal incontinence (18). Spinal cord injury is another major cause of neurological-related faecal incontinence (3). Damage to the spinal cord, often due to accidents or trauma, can disrupt the communication between the rectum and the brain, leading to impaired sensation, muscle control, and reflexes essential for maintaining continence (1). Depending on the level and extent of the injury, faecal incontinence can range from occasional leakage to complete loss of bowel control (1). Similarly, another cause of FI is multiple sclerosis (MS), which is an autoimmune disorder that affects the central nervous system by causing demyelination of nerves, leading to impaired nerve signals. This can result in disrupted bowel control and contribute to faecal incontinence (3). Furthermore, various neurological conditions, such as stroke, Parkinson's disease, or dementia, can affect the nerves involved in bowel control (1). The altered nerve function can disrupt the communication between the rectum and the brain, leading to impaired rectal sensation and coordination, ultimately resulting in faecal incontinence (1). In all these neurological disorders and nerve problems, the communication between the rectum, nerves, and brain is compromised, leading to deficits in bowel control.

### Other contributing factors

Conditions such as inflammatory bowel disease (IBD), irritable bowel syndrome (IBS), or infections can lead to chronic diarrhoea, contributing to the development of faecal incontinence (8). In addition, IBD, can also contribute to faecal incontinence due to the inflammation and damage to the intestinal lining, affecting rectal sensation and sphincter function (12).

Medications can play a significant role in causing or exacerbating faecal incontinence by influencing bowel motility, consistency, and nerve function. Laxatives, commonly used to treat constipation, can lead to faecal incontinence when overused or misused (8). Prolonged use of laxatives can result in chronic diarrhoea or loose stools, overwhelming the rectum and anal sphincters' capacity to hold stool properly, and leading to accidental leakage (19). On the other hand, antidiarrhoeal drugs, prescribed to manage diarrhoea, can also have unintended consequences for bowel control. While they can help control frequent bowel movements, they may cause stool to become more solid, leading to faecal impaction. Paradoxically, liquid stool may leak around the impacted mass, resulting in incontinence (19).

In addition, medications that alter nerve function can also impact bowel control and contribute to faecal incontinence (14). Some nerve-altering medications, such as those used to manage chronic pain or neurological conditions, can interfere with the normal signalling between the rectum and the brain, disrupting the coordination of bowel movements (14). As a result, individuals may experience diminished sensation or impaired voluntary control over bowel function, leading to faecal incontinence (14).

## Work up and diagnosis

The work-up for FI involves a comprehensive evaluation of the individual's medical history, physical examination, and diagnostic tests to identify the underlying physiological factors contributing to the condition and guide appropriate treatment strategies.

### History

*The Rome IV criteria, a classification system used to aid in the diagnosis of functional gastrointestinal disorders, are perhaps the most commonly employed criteria for diagnosing of FI. According to this classification, a confirmed diagnosis of FI involves recurrent involuntary passage of faecal matter in individuals aged ≥4 years, consistently experienced for at least 3 months. Interestingly, for research studies, symptoms should be evident for around 6 months with 2–4 instances of FI occurring within a 4-week span. It's worth noting that the Rome IV criteria have evolved from previous versions, but discussing their complete history is beyond the scope of this review* (22).

When taking a FI history, the duration and frequency of symptoms should be evaluated to understand the chronicity and pattern of incontinence. Characteristics of FI, such as the consistency and volume of stool, whether it is associated with urgency and identifying trigger factors including coughing, sneezing, or exertion are key in a FI history (23). Associated symptoms including diarrhoea, constipation, and bloating can also provide valuable clues to the underlying cause (3).

Past medical history should be explored to identify relevant medical conditions (inflammatory bowel disease, neurological disorders), surgical procedures (haemorrhoidectomy, fistulotomy, low anterior resection), obstetric history (primiparity, instrumental delivery, perineal tears) or treatment (pelvic radiation) that could potentially lead to FI (3, 18). Medication history should also be considered, as certain drugs can affect bowel function and contribute to FI such as opioids and laxatives (23). The impact of FI on the individual's daily life, including quality of life, social interactions, work, and emotional wellbeing is important to provide a comprehensive understanding of the overall burden of the condition on the patient (23).

### Physical examination

Physical examination should be performed to facilitate a reliable diagnosis whilst ensuring patient comfort (24). The perianal area should be inspected to reveal potential signs including irritation, deformities, haemorrhoids or previous surgical scars (15). A digital rectal examination (DRE) is performed to assess sphincter integrity, tone, and assess for the presence of rectocele, faecal impaction, or masses. Asking the patient to bear down during the DRE allows for the assessment of the function of pelvic floor muscles and puborectalis (3). Additionally, a vaginal examination should be performed where appropriate to assess for prolapse, rectocele, cystocele, or enterocele (15). Anoscopy may also be performed to directly visualise the anal canal and lower rectum to allow for the identification of anorectal lesions including fistulas, haemorrhoids, and proctitis (25).

### Severity scoring systems

Scoring systems play a crucial role in providing a standardised and quantitative assessment of FI and are valuable tools when used in conjunction with clinical evaluation and individualised assessment to comprehensively understand the condition and guide management. Several commonly used scoring systems have been developed to assess the severity and frequency of FI symptoms and the impact on patients quality of life. These include the Wexner Score (Cleveland Clinic Fecal Incontinence Severity Scoring System or CCIS) (26), Vaizey Score (St Mark's Incontinence Score) (27), and Faecal Incontinence Severity Index (FISI) (28). However, there is currently no globally accepted scoring algorithm for diagnosing FI (29).

The Wexner score is perhaps the most widely used scoring tool to assess FI, and has been shown to closely correlate with patient perception of symptoms and clinical assessment (26, 29, 30). However, the Wexner score gives equal weight to all symptoms potentially making assessment of sphincter impairment challenging and does not take into account faecal urgency (30). The Vaizey score was developed based on the Wexner score, with the addition of faecal urgency and constipating medications, however has been reported to be more difficult to understand due to the clinical language (27).

The American Society and of Colon and Rectal Surgeons Pelvic Floor Disorders Consortium recommended the routine use of “IMPACT” (Initial Measurement of Patient Reported Pelvic Floor Complaints Tool), which is a combination of the Wexner and Vaizey scores, whilst limiting the number of questions asked to patients (31).

FISI was developed using both surgeon and patient input and gives variable weights to symptoms based on the subjective experience (28). However, it excludes lifestyle impact, which is seen within other scoring tools and is a crucial component in understanding a patient's experience living with FI (30).

Despite a variety of scores being available many do not monitor symptoms of urgency (32), although the Vaizey score evaluates urgency it does not consider the frequency of urgency (33).

It has been shown that patients with the primary complaint of urgency FI report a significantly worse quality of life compared to those with passive FI as their primary complaint (34). Additionally, distinguishing between urgency and passive FI is important as functional differences can be found between the two groups which can then affect management (34). Passive incontinence is associated with those who have structural or functional damage to the IAS, whereas patients with damage to the EAS present with a primary complaint of urgency and frequent passage of stool (34).

The Low Anterior Resection Syndrome (LARS) score is primarily to assess LARS which is a collection of symptoms, including FI and frequency of urgency, which may impair quality of life in patients post complete or partial resection of the rectum (32). Bowel dysfunction and symptoms are seen in other anorectal complaints and as such the LARS score has previously been used to assess these symptoms, for example in women who have undergone surgery for endometriosis with and without bowel resection (35).

Notably, the Wexner score, the most widely used scoring system, is more likely to identify individuals with passive FI rather than those experiencing urgency FI (33). Recent literature on FI in women with previous obstetric injury suggests that combining the Wexner score with the LARS score can provide additional important information especially regarding urgency symptoms (33).

### Diagnostic tests

The treating physician may wish to perform luminal examination in the form of colonoscopy or similar to exclude malignancy, if “red flag” symptoms were elicited during history taking (36).

Depending on clinical findings and suspected underlying causes, additional diagnostic tests may be conducted to further evaluate FI. Most centres will opt for High Resolution Anorectal Manometry (HRAM), as per the American Gastroenterology guidelines (36). HRAM plays a crucial role in evaluating the function of the anorectal region, encompassing both motor and sensory aspects (37). Its use is pivotal in diagnosing FI, as it deepens the understanding of FI's underlying pathophysiology, thus enabling tailored therapies for individual patients. HRAM offers a dynamic analysis of anal sphincters and intraluminal rectal pressures, making it the most established method for objectively assessing various elements of anal and rectal function, including basal tone, contractility, recto-anal coordination, and reflex functions like recto-anal inhibitory reflex (37). Moreover, it measures rectal sensation thresholds, a valuable predictor of response to biofeedback training (38).

When contemplating surgery for individuals with incontinence and diminished anal pressures, it's important to assess the structural integrity of the anal sphincters, rectal wall, and the puborectalis muscle region. This can be accomplished using either endoscopic anal ultrasound (EAUS) or MRI (36). While these tests share some commonalities in identifying issues like scars, defects, or thinning, each offers unique diagnostic capabilities. For instance, ultrasound excels at detecting tears in the IAS, whereas MRI is more adept at identifying atrophy in the EAS (28, 39). Furthermore, distinguishing between an EAS tear and a scar is more accurate with MRI (36).

EAUS is the gold standard examination for assessing the anal sphincter integrity. It is well tolerated and widely available (8). Nevertheless, its efficacy is contingent upon the operator's proficiency, and there is ongoing debate regarding its sensitivity in accurately identifying anal sphincter integrity (40). MRI is less easily accessible, more costly, and poses limitations in patients with defibrillators, metal implants, or those who experience claustrophobia. In the absence of these concerns, initiating the diagnostic process with ultrasound and subsequently advancing to MRI is deemed appropriate (8).

Additional tests may include defecography, endoanal MRI or Pudendal nerve terminal motor latency (PNTML) (15).

Defecography is a diagnostic procedure used to evaluate the function and anatomy of the pelvic floor during defecation. It provides valuable insights into the mechanisms of stool evacuation and can help diagnose various conditions related to the pelvic floor, such as rectal prolapse, rectoceles, intussusception, and obstructive defecation syndrome (39).

Fluoroscopic x-ray defecography involves the patient ingesting a contrast medium, typically a barium-based solution, before having x-rays taken while they expel the contrast medium during defecation. This allows real-time visualisation of the movement of the pelvic structures and the rectal contents. Fluoroscopic defecography is advantageous for its ability to capture dynamic images and assess the coordination and mechanics of the pelvic floor muscles during evacuation (41).

On the other hand, magnetic resonance imaging (MRI) defecography employs advanced imaging technology to create detailed, high-resolution images of the pelvic structures and their movement during defecation. MRI defecography provides a more comprehensive definition of all 3 compartments (42, 43).

The choice between these two imaging modalities depends on factors such as the specific clinical question, patient comfort, and the availability of equipment. Fluoroscopic x-ray defecography offers dynamic insights, while MRI defecography provides precise anatomical detail. Both techniques play a crucial role in diagnosing and understanding pelvic floor dysfunction, helping guide appropriate treatment strategies (44).

PNTML (Pudendal Nerve Terminal Motor Latency) is a test used to assess the time it takes for an electrical signal to travel along the pudendal nerve from the ischial spine to the anal verge. This test aids in evaluating the neuromuscular integrity of the pelvic floor. The pudendal nerve is stimulated near the ischial spine through the anus, and the time between nerve stimulation and muscle response is measured. Any impairment in the neuromuscular unit can lead to a lengthened latency period (45).

Numerous studies have revealed that PNTML prolongation is observed after uncomplicated vaginal delivery. Although PNTML values tend to approach the reference range three months postpartum, Tetzschner et al. discovered that a significant and lasting PNTML prolongation persisted in incontinent women compared to continent women at the three-month postpartum mark. Furthermore, abnormal PNTML was identified as the sole predictor for the development of anal incontinence 2–4 years postpartum in women who had experienced anal sphincter rupture (46).

Although initial studies have demonstrated prolonged PNTMLs in individuals with idiopathic faecal incontinence compared to healthy controls (47), subsequent studies have also identified PNTML prolongation in other conditions including chronic constipation and proctalgia (48). In addition, half of those patients with prolonged PNTML exhibited normal anal canal squeeze pressures (48). The lack of association between PNTML prolongation and decreased anal canal squeeze pressures has been demonstrated in further studies (49, 50) Failing to control for age could contribute to poor correlation as PNTML has been shown to increase with age, independent of continence status (49). Furthermore, PNTML measures only the conduction time of the fastest fibers in the pudendal nerve, with the potential for normal conduction times despite nerve damage if some fast-conducting fibers remain intact (49).

The poor sensitivity and specificity of PNTML in detecting EAS muscle weakness resulting from pudendal nerve damage remains a concern (51). Moreover, it is an operator dependent test with a poorly defined upper limit given the large variation in healthy individuals. As a result its clinical utility is limited (38) and PNTML should considered primarily of research interest only (24).

### Role of multidisciplinary team (MDT)

The complexity of FI lends itself to an MDT approach with studies confirming effectiveness of the MDT in enhancing patient satisfaction and promoting greater adherence to treatment (52, 53). A typical MDT could include specialist surgeons (colorectal, gynaecology, urology) physiotherapists, clinical scientists, specialist nurses and radiologists for example (54). The MDT facilitates discussions regarding patients where current treatment is ineffective, enables the review of imaging and results, and allows for the consideration of additional non operative or surgical procedures including joint procedures in individuals with multicompartment prolapse (54). The MDT also provides the opportunity for less specialised surgeons to attend, either face to face or via videoconferencing to gather support and increased knowledge from the established expert network (55).

## Treatment

Management of faecal incontinence (FI) is complex and challenging, therefore a holistic approach that gives careful consideration of not only the aetiology but of a patient's psychosocial and medical background is required. Currently there is not an abundance of high-quality evidence for the management of FI and successful outcomes appear dependent on the interplay of many factors. The international consensus is that conservative interventions are recommended initially. Adopting this approach first mainly aims at reducing risk factors for FI and avoiding morbidity associated with more invasive interventions (19).

### Non operative management

Conservative management includes lifestyle changes mainly weight reduction, smoking cessation, dietary modification, pelvic floor physiotherapy, bowel retraining, medication and environmental review. Research has shown that dedicated nurse specialist clinics alone can improve symptoms (56). They offer education and support into establishing a consistent routine. When appropriate and relevant, the involvement of caregivers is essential in maintaining any positive outcomes.

Dietary modification that involves increasing fibre and reducing fluid intake to optimise stool consistency is advised (except for patients with FI related to constipation) (57). Patients are recommended to maintain incontinence journals to identify possible triggers. It is advised to avoid the consumption of caffeine, particularly coffee, due to its recognised laxative properties, as well as lactose, excessive vitamin C, magnesium phosphorus, artificial sweeteners, alcohol and chilli (31, 58).

Physiotherapy was found to be a fundamental component in the treatment of individuals with faecal incontinence (FI) and enhancing their overall quality of life (59). The benefits in primary prevention were also described. Medication review is essential to rule out side effects from their regular prescription medications which may be contributing to urgency or diarrhoea such as Donepezil and Rivastigmine, calcium channel agonists and metformin (31, 58) Whilst awaiting referral and review by a specialist, short term management such as foam plugs, and RADAR Keys can be offered (58).

Medical management involves more specialised interventions such as drug prescription, rectal irrigation, physiotherapy and biofeedback. Pharmacological interventions aim to decelerate colonic motility and enhance stool consistency by reducing intestinal fluid secretion, promoting absorption, and minimising sphincter relaxation. The addition of fibre in treatment can effectively manage variations in stool consistency and is recommended by National Institute of Clinical Excellence (NICE) and the International continence society (ICS) (60). In a randomised controlled trial involving 39 patients, the group receiving fibre supplementation experienced a decrease in the percentage of incontinent stools to less than half compared to the placebo group, demonstrating an improvement in stool consistency (61). Anti-diarrhoeal medications are suggested for FI with pre-existing diarrhoea (31). The initial treatment is loperamide hydrochloride. For patients who require additional options or cannot tolerate loperamide, codeine phosphate may be offered. Alternatively, co-phenotrope can be considered for those who are intolerant to loperamide (58). Laxatives are only recommended for those with faecal loading (58). There is mixed guidelines regarding Colestyramine, the ASCRS recommends in those with a history of cholecystectomy or ileocolonic resection, but other guidelines do not yet include it (60).

Biofeedback of different modalities (such as EMG and manometric biofeedback) coupled with pelvic floor exercises or electrostimulation can enhance treatment outcome (62). As well as individually, evidence has described how a combination of therapies such as medications, biofeedback and pelvic floor exercises can lead to an improvement in symptoms (62).

Once less invasive measures have failed bowel management programmes are tried, training patients to facilitate emptying with enemas and suppositories, or more complex regimens using trans anal irrigation (TAI), ensuring no absolute contraindications prior to doing so. TAI requires specific devices and education on how it should be administered and should only be done if suitable. A recent systematic review found for a cohort of patients improvement in bowel function and quality of life (62). TAI is indicated prior to surgery but dependent on patient preference, it is most effective in those with faecal loading or spinal or neurological disease or injury (58, 60).

#### Role of primary and secondary care

The role of primary care in managing faecal incontinence is contingent on local resources. Initial assessment and management should commence in the community, emphasising dietary and fluid modifications for stool consistency and regular bowel emptying. Other interventions might include addressing home toilet accessibility, providing necessary equipment, and reviewing regular medications. Depending on the underlying cause, loperamide or laxatives may be considered in the community as appropriate, along with the provision of radar keys, anal plugs, skin care guidance, barrier products, and disposable gloves (58).

Signposting routes to access emotional and psychological wellbeing may be provided by either primary or secondary care as appropriate depending on access (58).

More specialised services like pelvic floor muscle retraining, physiotherapy, rectal irrigation, biofeedback and specialised dietary assessment and management are usually offered within secondary care environment. Individuals being considered for surgery should be evaluated in secondary care (58).

#### Surgical management

If non-operative measures are ineffective, surgical options can be offered. Obvious structural deformities such as full thickness rectal prolapse or fistula must be repaired first (14). Surgical approach then aims at restoration of anatomy, improvement of sphincteric complex functioning or lastly diversion. A summary of previous, current and future surgical options can be seen in Table 1.

**Table 1:** Summary of current availability of surgical options.

Sphincteroplasty	Available
SNS	Available
Antegrade Colonic Enema	Available
Artificial Bowel Sphincter Implantation	In phase of study
Magnetic Anal Sphincter Implantation	Not available
Biomaterial injectables	Available
	New materials in phase of study
Dynamic Gracioplasty	Not available
Adynamic Gracioplasty	Available—selected patients
Theirsch wire	Available in rectal prolapse—palliative approach
Pelvic sling	Available in rectal prolapse
Colostomy (faecal diversion)	Available—Final option

### Biomaterial injectables

A distinct method for enhancing the sphincter complex is Injection or implantation of bulking agents. The rationale is to achieve increased passive outlet resistance by adding volume to the anal canal or perianal tissues. Various techniques and materials have been used for this purpose. Patient selection remains undefined but could encompass those with mild passive incontinence due to internal anal sphincter weakness or postsurgical deformities altering the anal canal shape (14).

A systematic review encompassing 16 studies (none of which was randomised) involving 420 patients examined conventional injectables (Carbon, Teflon or silicon, collagen, autologous fat) revealing limited evidence for their efficacy in passive faecal incontinence. Only 2 studies achieved over 50% improvement, while others reported 15%–50% improvements at long-term follow-ups. Complications affected up to 10%, with side effects reaching 12% (63). Newer materials include non-animal stabilised hyaluronic acid/dextranomer. They gained popularity amongst both specialists and general practitioners, with an outpatient/office-based injection approach gained momentum in 2011 after a randomised, placebo-controlled trial involving 206 patients showed a greater than 50% improvement in 53.2% vs. 30.7% in the intervention vs. sham groups, respectively (64). However, the intervention did not stand the test of time as complete continence rate at 6 months was 6%. Selection criteria uncertainty and durability and cost concerns, impeded the technique's widespread adoption (65).

Two recent strategies include the implantation of self-expandable hyexpan (polyacrylonitrile) prosthesis via an applicator gun (66) and the use of stem cells (67). Although they have been evaluated through small cohort studies (67–69), results from larger randomised trials are awaited.

### Sphincter repair

If a segmental sphincteric defect is identified, normally related to obstetric injury which involves the full length of the EAS and a defect of 90 degrees, or greater than, sphincteroplasty can be considered to directly repair the injured muscles (42). Good to excellent results have been observed in around 85% of patients in the short term. However, long term positive outcomes are rarely maintained and only 10%–14% of patients with sphincteroplasty exhibited sustained improvement in function at 5 years (31). Given this, the patient must be properly counselled prior to such procedure (31). The efficacy of sphincteroplasty has come under scrutiny, especially in women experiencing faecal incontinence decades after obstetric trauma so careful consideration is needed in these patients (31).

### Sacral neuromodulation

Enhancement of the sphincter can occur with the placement of a sacral (SNS) or a percutaneous tibial nerve stimulator (PTNS) (14). Over the last decades, SNS has brought about a transformative shift in the management of faecal incontinence (70). The technique involves two outpatient procedures under light anaesthesia. In the initial procedure, a 4-point electrode is placed at the sacral root S3 and connected to a temporary external stimulation device. If the patient responds positively during the subsequent 2-week trial period, a permanent implantation of the stimulator device similar to a pacemaker is carried out in the second surgery; otherwise, the electrode is removed. While the exact mechanism remains partially understood, SNS is thought to reactivate a dysfunctional pelvic floor and receptor pathway while also engaging the brain's afferent pathway related to continence (71, 72).

Regardless of the precise mechanism, the outcomes are impressive, with over two-thirds of patients experiencing over 50% improvement, leading to permanent stimulator implantation (65). This positive impact consistently maintained, both immediately and over the long term. After the permanent implantation, 86%–87% of patients reported over 50% improvement, and around 40% achieved complete control, with these successes lasting beyond 3–5 years (65, 73). The complication rate is relatively low, with infection and electrode dislocation being the most common, occurring at rates of 3% and 12%, respectively (45, 74). However, subsequent interventions for revision or device replacement (due to battery life) are required in 19%–36% of cases (74, 75). Recent advancements have brought about the utilisation of rechargeable batteries with a claimed lifespan of more than 20 years, requiring recharging every 6 to 10 months (76).

NICE recommends SNS when sphincter surgery is deemed inappropriate for example where there is no defect, or there is sphincter disruption or sphincter defect with atrophy, denervation, a small defect, absence of voluntary contraction or poor quality muscle (77). However, the ASCRS recommends SNS as first line treatment for those with or without sphincter defects (31, 60).

Beyond sphincter modifications, a variety of methods of replacing the anal sphincter have been attempted, some more effectively than others. These approaches are geared towards either restoring or enhancing the functionality of the anal sphincter muscles (14). They can be categorised to dynamic and non-dynamic techniques (14).

### Dynamic sphincter replacement

Artificial Bowel Sphincter Implantation: This method involves surgically implanting an artificial device to mimic the role of the natural anal sphincter. This artificial sphincter allows for dynamic control of bowel movements. However, its use has been restricted due to the risk of infection and potential long-term device-related complications (59).

Magnetic Anal Sphincter Implantation: In this approach, a magnetic ring is implanted around the anal canal. By creating passive resistance, the device contributes to controlling faecal continence (65). While initial feasibility studies showed promise, due to high rates of significant events, complications and explanation, both this and dynamic graciloplasty are no longer available (78).

Dynamic Graciloplasty: This technique utilises the gracilis muscle harvested from the thigh, wrapping it around the anal canal. Although voluntary control of this muscle is limited, an implanted pulse generator can transform its properties over time, leading to improved faecal control (79). For similar reasons as for magnetic sphincters this technique is no longer popular or available (78).

### Nondynamic sphincter

Thiersch and Similar Procedures: Encircling materials are placed around the anal canal to narrow it and heighten passive resistance. While the aim is to enhance control over bowel movements, limited data exist to support its effectiveness (14). It is normally reserved for patients who are in a debilitated state, with the primary goal being symptom palliation (72).

Non-dynamic Graciloplasty/Gluteoplasty: This technique involves wrapping muscles like the gracilis or gluteus around the anal canal without stimulation. However, its application is limited due to the heightened risk of complications and limited functional improvement (80). A systematic review encompassing 14 studies involving 450 patients identified similar functional results between dynamic and adynamic graciloplasty, but with a higher risk of reoperation and complications in the dynamic graciloplasty. Consequently, non-dynamic graciloplasty is the preferred approach (81).

Pelvic Floor Repairs/Sling: Addressing pelvic floor support to restore anorectal angles and improve faecal control is the focus of this approach. Recent attention has been directed towards an investigational trans-obturator posterior anal sling system. Results from clinical trials have shown promising outcomes, including treatment success and enhanced continence rates (14).

These replacement techniques offer diverse strategies for tackling faecal incontinence, accommodating varying patient needs and conditions. The selection of the most suitable technique hinges on factors such as the specific condition of the patient, expected outcomes, and potential risks associated with the procedure (14).

Percutaneous tibial nerve stimulation (PTNS) is another nerve stimulation method employed in the management of faecal incontinence (82). Through the use of either transcutaneous or percutaneous electrodes, the posterior tibial nerve is stimulated during sessions lasting around 30 min, carried out over a period of at least 3 months (82). While the specific benefits and mechanism of action of tibial nerve stimulation are less straightforward and remain somewhat elusive, it is believed to influence faecal control by activating the central nervous system and supra-sacral neural centres via the afferent fibres of the peripheral nervous system. Given that the posterior tibial nerve originates from lumbar and sacral nerve ventral branches, a similar response as seen with SNS is anticipated (83).

Despite the anticipation, favourable results were not noted in CONFIDeNT, a double-blind, multicentre, pragmatic, randomised controlled trial conducted in 17 UK hospital units specialising in faecal incontinence management (84). Participants with substantial faecal incontinence not responding to conservative treatments were randomly assigned to receive either percutaneous tibial nerve stimulation (PTNS) or sham stimulation for 12 weeks. The primary outcome was a 50% reduction in weekly faecal incontinence episodes (84). Among the 227 eligible patients assigned to PTNS (*n* = 115) or sham (*n* = 112), 38% in the PTNS group and 31% in the sham group met the primary outcome. No serious treatment-related adverse events occurred. PTNS did not significantly outperform sham stimulation in this 12-week trial (84).

Another surgical option is ACE (antegrade colonic enema) mainly utilised in paediatrics or those with colonic motility disorders (85, 86). Initially introduced by Malone et al. in 1990, subsequent refinements to the ACE procedure have resulted in well-established laparoscopic techniques that are employed for children experiencing persistent constipation issues (85, 86). The treatment encompasses flushing colonic contents in a forward direction through a surgically formed catheterisable channel in the abdominal wall (85, 86). This is performed most commonly either through an appendicostomy or a caecostomy (87). Studies have shown this to be effective for children with refractory FI or constipation (87).

Finally, faecal diversion through the establishment of a colostomy or ileostomy represents a definitive solution for managing faecal incontinence. While an ileostomy might be considered for patients with colonic transit irregularities, the colostomy is the standard ostomy approach employed in treating faecal incontinence (88). Although a colostomy carries short and long-term risks, it is a viable, secure and efficient intervention for severe faecal incontinence (88).

Whilst patients often harbour apprehensions about permanent colostomy due to concerns over its management, self-image, and social interactions; when individuals who underwent colostomy for faecal incontinence were surveyed, their overall quality of life and faecal incontinence-specific quality of life scores were higher compared to those of other individuals with faecal incontinence (89). Furthermore, a separate study revealed that patients generally expressed high levels of satisfaction with their stomas for faecal incontinence, with over 80% indicating they would willingly undergo the procedure again (90). Colostomy offers the most cost-effective approach in terms of quality-adjusted life years (91).

## Conclusion

FI is a complex and multifaceted medical condition, often posing a diagnostic and management challenge for the generalist as well as the specialised colorectal surgeon. This narrative review aims to give a comprehensive overview of the pathophysiology, the diagnostic mechanisms and the treatment options, to assist the generalist to manage FI.

## Data Availability

The original contributions presented in the study are included in the article/Supplementary Material, further inquiries can be directed to the corresponding author.
